# 2-(Trifluoro­meth­yl)benzoic acid

**DOI:** 10.1107/S1600536811009597

**Published:** 2011-03-19

**Authors:** Richard Betz, Thomas Gerber

**Affiliations:** a Nelson Mandela Metropolitan University, Summerstrand Campus, Department of Chemistry, University Way, Summerstrand, PO Box 77000, Port Elizabeth 6031, South Africa

## Abstract

In the title compound, C_8_H_5_F_3_O_2_, a halogenated derivative of benzoic acid, the carboxyl group is tilted by 16.8 (3)° with respect to the plane of the aromatic ring. In the crystal, O—H⋯O hydrogen bonding gives rise to carb­oxy­lic acid dimers, which are further connected into double chains along [1,1/4,1] by C—H⋯O contacts. C—H⋯F and C—F⋯π contacts are also observed.

## Related literature

For the crystal structure of benzoic acid using X-ray diffraction, see Bruno & Randaccio (1980 ▶). For the crystal structure of benzoic acid applying neutron radiation, see Wilson *et al.* (1996 ▶), and of *ortho*-fluoro­benzoic acid, see Krausse & Dunken (1966 ▶). For the crystal structure of *ortho*-chloro­benzoic acid, see Ferguson & Sim (1961 ▶); Polito *et al.* (2008 ▶). For graph-set analysis of hydrogen bonds, see: Etter *et al.* (1990 ▶); Bernstein *et al.* (1995 ▶).
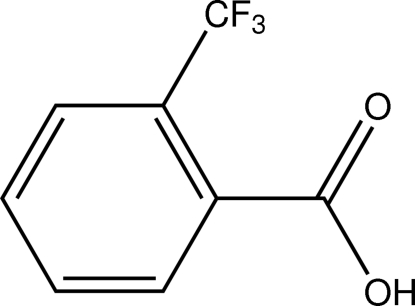


## Experimental

### 

#### Crystal data

C_8_H_5_F_3_O_2_
*M* *_r_* = 190.12
Monoclinic, 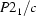

*a* = 4.8816 (3) Å
*b* = 20.6948 (14) Å
*c* = 7.9697 (5) Å
β = 109.544 (4)°
*V* = 758.74 (8) Å^3^
*Z* = 4
Mo *K*α radiation
μ = 0.17 mm^−1^
*T* = 200 K
0.50 × 0.50 × 0.09 mm

#### Data collection

Bruker APEXII CCD diffractometer
7115 measured reflections
1889 independent reflections
1548 reflections with *I* > 2σ(*I*)
*R*_int_ = 0.048

#### Refinement

*R*[*F* ^2^ > 2σ(*F* ^2^)] = 0.049
*wR*(*F* ^2^) = 0.113
*S* = 1.06
1889 reflections
119 parameters
H-atom parameters constrained
Δρ_max_ = 0.30 e Å^−3^
Δρ_min_ = −0.33 e Å^−3^

### 

Data collection: *APEX2* (Bruker, 2010 ▶); cell refinement: *SAINT* (Bruker, 2010 ▶); data reduction: *SAINT*; program(s) used to solve structure: *SHELXS97* (Sheldrick, 2008 ▶); program(s) used to refine structure: *SHELXL97* (Sheldrick, 2008 ▶); molecular graphics: *ORTEP-3* (Farrugia, 1997 ▶) and *Mercury* (Macrae *et al.*, 2006 ▶); software used to prepare material for publication: *SHELXL97* and *PLATON* (Spek, 2009 ▶).

## Supplementary Material

Crystal structure: contains datablocks I, global. DOI: 10.1107/S1600536811009597/fl2339sup1.cif

Structure factors: contains datablocks I. DOI: 10.1107/S1600536811009597/fl2339Isup2.hkl

Additional supplementary materials:  crystallographic information; 3D view; checkCIF report

## Figures and Tables

**Table 1:** Hydrogen-bond geometry (Å, °) *Cg* is the centroid of the C2–C7 ring.

*D*—H⋯*A*	*D*—H	H⋯*A*	*D*⋯*A*	*D*—H⋯*A*
O1—H1⋯O2^i^	0.84	1.81	2.6459 (19)	173
C6—H6⋯O2^ii^	0.95	2.66	3.590 (3)	167
C6—H6⋯F3^ii^	0.95	2.63	3.303 (3)	128
C7—H7⋯O1^iii^	0.95	2.66	3.411 (2)	137
C8—F1⋯*Cg*^iv^	1.34 (1)	3.48 (1)	4.806 (2)	170 (1)

Symmetry codes: (i) 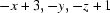; (ii) 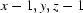; (iii) 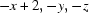; (iv) 
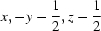
.

**Fig. 1.**
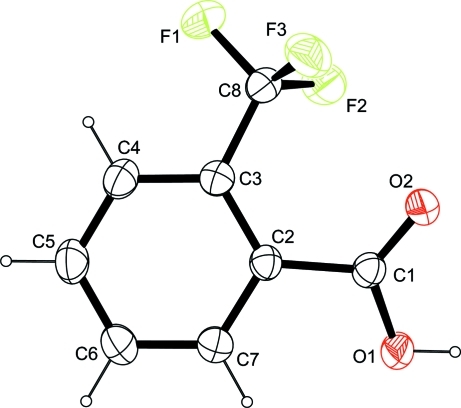
The molecular structure of the title compound, with atom labels and anisotropic displacement ellipsoids (drawn at 50% probability level).

**Fig. 2.**
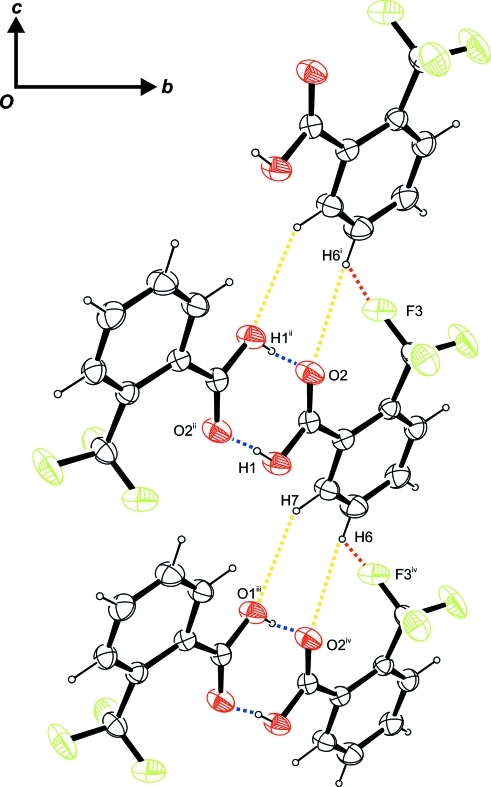
Intermolecular contacts, viewed along [-1 0 0]. Hydrogen bonds are depicted with blue dashed lines, C–H···O contacts with yellow dashed lines and C–H···F contacts with red dashed lines. Symmetry operators: ix + 1, y, z + 1; ii -x + 3, -y, -z + 1; iii -x + 2, -y, -z; ivx - 1, y, z - 1.

**Fig. 3.**
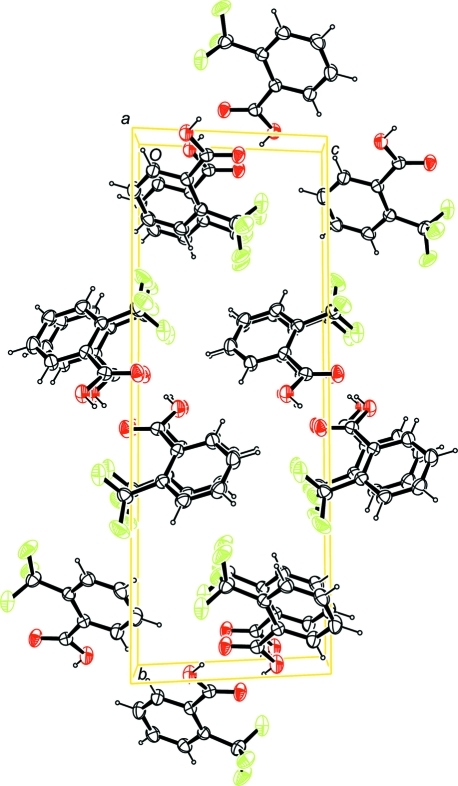
Molecular packing of the title compound, viewed along [-1 0 0] (anisotropic displacement ellipsoids drawn at 50% probability level).

## supplementary crystallographic information

### Comment

Benzoic acid has found widespread use as a ligand in coordination chemistry for a variety of transition metals and elements from the s- and *p*-block of the periodic system of the elements. It can act as a neutral or – upon deprotonation – an anionic ligand and serve as mono- or bidentate ligand. By varying the substituents on the phenyl moiety, the acidity of the carboxylic acid group can be fine-tuned. Particular interest rests in benzoic acid derivatives showing an asymmetric pattern of substituents on the aromatic moiety due to different possible orientations of the ligand in coordination compounds and the possible formation of stereoisomeric products. At the beginning of a comprehensive study aimed at rationalizing the coordination behaviour of various benzoic acid derivatives towards a number of transition metals in dependence of the *p*H value of the reaction batches it seemed interesting to determine the crystal structure of the title compound to enable comparative studies. The crystal structure of unsubstituted benzoic acid (Bruno & Randaccio (1980); Wilson *et al.* (1996)) as well as the crystal structures of benzoic acid derivatives bearing a halogen atom in an *ortho*-position to the carboxylic acid group (Krausse & Dunken (1966); Ferguson & Sim (1961); Polito *et al.* (2008)) are apparent in the literature.

C–C–C angles within the phenyl ring span a range of 118 ° to 121 ° with the biggest as well as the smallest angle found on both C-atoms in the *ortho*-position to the C-atom bearing the carboxylic acid group. The latter one is found on the carbon atom bearing the trifluoromethyl group (Fig. 1).

The carboxylic acid group is slightly tilted with respect to the plane of the aromatic moiety. The least-squares planes defined by their respective atoms enclose an angle of 16.81 (26) °.

In the crystal structure, hydrogen bonds between the carboxylic acid groups of two molecules give rise to centrosymmetric dimers. These are further connected into double chains along [1 1/4 1] by C–H···O contacts whose range falls slightly below the sum of van-der-Waals radii of the corresponding atoms (Fig. 2). The latter contacts can be observed between the hydrogen atoms bonded to the carbon atoms in the *ortho*- as well as the *meta*-position to the carboxylic acid group and have the alcoholic as well as the carbonylic O-atom as acceptor. Additionally, the H-atom in the  *meta*-position to the carboxylic acid group forms a contact to one of the fluorine atoms of the trifluoromethyl group. In terms of graph-set analysis (Etter *et al.* (1990); Bernstein *et al.* (1995).), the descriptor for the classical hydrogen bonds building the centrosymmetric dimers is *R*^2^_2_(8) on the unitary level while the C–H···O-contacts necessitate a *C*^1^_1_(6)*R*^2^_2_(10) descriptor on the same level. No π-stacking is obvious in the compound, however, a C–F···*C*_g_ interaction (F···*C*_g_: 3.4803 (17) Å) can be observed.

The packing of the compound is shown in Figure 3.

### Experimental

The compound was obtained commercially (fluorochem). Crystals suitable for the X-ray diffraction study were grown from an aqueous solution of the compound.

### Refinement

Carbon-bound H-atoms were placed in calculated positions (C—H 0.95 Å) and were included in the refinement in the riding model approximation, with *U*(H) set to 1.2*U*_eq_(C). The H atom of the carboxylic acid group was allowed to rotate with a fixed angle around the C—O bond to best fit the experimental electron density (HFIX 147 in the *SHELX* program suite (Sheldrick, 2008)).

### Crystal data

**Table:**

C_8_H_5_F_3_O_2_	*F*(000) = 384
*M**_r_* = 190.12	*D*_x_ = 1.664 Mg m^−^^3^
Monoclinic, *P*2_1_/*c*	Mo *K*α radiation, λ = 0.71073 Å
Hall symbol: -P 2ybc	Cell parameters from 4165 reflections
*a* = 4.8816 (3) Å	θ = 2.7–28.3°
*b* = 20.6948 (14) Å	µ = 0.17 mm^−^^1^
*c* = 7.9697 (5) Å	*T* = 200 K
β = 109.544 (4)°	Platelet, colourless
*V* = 758.74 (8) Å^3^	0.50 × 0.50 × 0.09 mm
*Z* = 4	

### Data collection

**Table:**

Bruker APEXII CCD diffractometer	1548 reflections with *I* > 2σ(*I*)
Radiation source: fine-focus sealed tube	*R*_int_ = 0.048
graphite	θ_max_ = 28.3°, θ_min_ = 3.4°
φ and ω scans	*h* = −6→6
7115 measured reflections	*k* = −27→27
1889 independent reflections	*l* = −10→10

### Refinement

**Table:**

Refinement on *F*^2^	Primary atom site location: structure-invariant direct methods
Least-squares matrix: full	Secondary atom site location: difference Fourier map
*R*[*F*^2^ > 2σ(*F*^2^)] = 0.049	Hydrogen site location: inferred from neighbouring sites
*wR*(*F*^2^) = 0.113	H-atom parameters constrained
*S* = 1.06	*w* = 1/[σ^2^(*F*_o_^2^) + (0.0324*P*)^2^ + 0.680*P*] where *P* = (*F*_o_^2^ + 2*F*_c_^2^)/3
1889 reflections	(Δ/σ)_max_ < 0.001
119 parameters	Δρ_max_ = 0.30 e Å^−^^3^
0 restraints	Δρ_min_ = −0.33 e Å^−^^3^

### Fractional atomic coordinates and isotropic or equivalent isotropic displacement parameters (Å^2^)

**Table:**

	*x*	*y*	*z*	*U*_iso_*/*U*_eq_	
F1	0.8290 (3)	0.22541 (7)	0.5490 (2)	0.0602 (5)	
F2	1.2257 (3)	0.17857 (6)	0.56593 (18)	0.0453 (3)	
F3	0.9086 (3)	0.12968 (7)	0.65393 (16)	0.0455 (3)	
O1	1.2631 (3)	0.01372 (7)	0.27504 (18)	0.0366 (3)	
H1	1.4044	−0.0080	0.3397	0.055*	
O2	1.2658 (3)	0.05058 (7)	0.53681 (17)	0.0368 (3)	
C1	1.1610 (4)	0.04997 (8)	0.3755 (2)	0.0262 (4)	
C2	0.9025 (4)	0.08893 (8)	0.2720 (2)	0.0252 (3)	
C3	0.7956 (4)	0.14205 (8)	0.3418 (2)	0.0264 (4)	
C4	0.5485 (4)	0.17387 (9)	0.2361 (3)	0.0324 (4)	
H4	0.4766	0.2097	0.2833	0.039*	
C5	0.4046 (5)	0.15424 (10)	0.0626 (3)	0.0366 (4)	
H5	0.2336	0.1761	−0.0078	0.044*	
C6	0.5102 (5)	0.10294 (11)	−0.0075 (3)	0.0381 (5)	
H6	0.4137	0.0897	−0.1268	0.046*	
C7	0.7571 (4)	0.07086 (10)	0.0963 (2)	0.0329 (4)	
H7	0.8292	0.0357	0.0467	0.040*	
C8	0.9405 (4)	0.16805 (9)	0.5273 (3)	0.0334 (4)	

### Atomic displacement parameters (Å^2^)

**Table:**

	*U*^11^	*U*^22^	*U*^33^	*U*^12^	*U*^13^	*U*^23^
F1	0.0651 (10)	0.0483 (8)	0.0547 (8)	0.0222 (7)	0.0035 (7)	−0.0228 (7)
F2	0.0332 (7)	0.0462 (7)	0.0510 (7)	−0.0084 (5)	0.0069 (6)	−0.0134 (6)
F3	0.0422 (7)	0.0658 (9)	0.0285 (6)	0.0038 (6)	0.0119 (5)	−0.0006 (6)
O1	0.0363 (8)	0.0448 (8)	0.0270 (6)	0.0172 (6)	0.0084 (6)	−0.0008 (6)
O2	0.0342 (8)	0.0455 (8)	0.0255 (6)	0.0146 (6)	0.0034 (5)	−0.0036 (6)
C1	0.0231 (8)	0.0258 (8)	0.0293 (8)	0.0008 (6)	0.0083 (7)	−0.0006 (7)
C2	0.0227 (8)	0.0275 (8)	0.0254 (8)	0.0014 (6)	0.0081 (6)	0.0015 (7)
C3	0.0264 (9)	0.0269 (8)	0.0269 (8)	0.0000 (7)	0.0101 (7)	0.0009 (7)
C4	0.0331 (10)	0.0297 (9)	0.0352 (9)	0.0070 (7)	0.0126 (8)	0.0039 (8)
C5	0.0328 (10)	0.0389 (10)	0.0342 (10)	0.0091 (8)	0.0062 (8)	0.0081 (8)
C6	0.0358 (11)	0.0469 (11)	0.0266 (9)	0.0061 (9)	0.0036 (8)	−0.0002 (8)
C7	0.0331 (10)	0.0372 (10)	0.0270 (9)	0.0062 (8)	0.0080 (7)	−0.0023 (7)
C8	0.0300 (10)	0.0343 (10)	0.0341 (10)	0.0052 (8)	0.0084 (8)	−0.0065 (8)

### Geometric parameters (Å, °)

**Table:**

F1—C8	1.341 (2)	C3—C4	1.385 (3)
F2—C8	1.339 (2)	C3—C8	1.509 (3)
F3—C8	1.333 (2)	C4—C5	1.386 (3)
O1—C1	1.311 (2)	C4—H4	0.9500
O1—H1	0.8400	C5—C6	1.378 (3)
O2—C1	1.214 (2)	C5—H5	0.9500
C1—C2	1.492 (2)	C6—C7	1.382 (3)
C2—C7	1.393 (2)	C6—H6	0.9500
C2—C3	1.409 (2)	C7—H7	0.9500
			
C1—O1—H1	109.5	C6—C5—H5	120.1
O2—C1—O1	122.74 (16)	C4—C5—H5	120.1
O2—C1—C2	123.96 (16)	C5—C6—C7	119.81 (19)
O1—C1—C2	113.29 (15)	C5—C6—H6	120.1
C7—C2—C3	118.36 (16)	C7—C6—H6	120.1
C7—C2—C1	117.59 (16)	C6—C7—C2	121.46 (18)
C3—C2—C1	124.05 (16)	C6—C7—H7	119.3
C4—C3—C2	119.55 (17)	C2—C7—H7	119.3
C4—C3—C8	117.00 (16)	F3—C8—F2	107.39 (16)
C2—C3—C8	123.43 (16)	F3—C8—F1	105.94 (17)
C3—C4—C5	121.01 (18)	F2—C8—F1	105.20 (16)
C3—C4—H4	119.5	F3—C8—C3	113.20 (16)
C5—C4—H4	119.5	F2—C8—C3	113.19 (16)
C6—C5—C4	119.79 (18)	F1—C8—C3	111.35 (16)
			
O2—C1—C2—C7	−162.52 (18)	C4—C5—C6—C7	0.8 (3)
O1—C1—C2—C7	16.4 (2)	C5—C6—C7—C2	0.4 (3)
O2—C1—C2—C3	16.7 (3)	C3—C2—C7—C6	−1.3 (3)
O1—C1—C2—C3	−164.37 (17)	C1—C2—C7—C6	177.90 (19)
C7—C2—C3—C4	1.1 (3)	C4—C3—C8—F3	108.95 (19)
C1—C2—C3—C4	−178.06 (17)	C2—C3—C8—F3	−72.5 (2)
C7—C2—C3—C8	−177.38 (18)	C4—C3—C8—F2	−128.55 (19)
C1—C2—C3—C8	3.4 (3)	C2—C3—C8—F2	50.0 (2)
C2—C3—C4—C5	0.0 (3)	C4—C3—C8—F1	−10.3 (3)
C8—C3—C4—C5	178.62 (18)	C2—C3—C8—F1	168.25 (18)
C3—C4—C5—C6	−1.0 (3)		

### Hydrogen-bond geometry (Å, °)

**Table:**

Cg is the centroid of the C2–C7 ring.

**Table:**

*D*—H···*A*	*D*—H	H···*A*	*D*···*A*	*D*—H···*A*
O1—H1···O2^i^	0.84	1.81	2.6459 (19)	173
C6—H6···O2^ii^	0.95	2.66	3.590 (3)	167
C6—H6···F3^ii^	0.95	2.63	3.303 (3)	128
C7—H7···O1^iii^	0.95	2.66	3.411 (2)	137
C8—F1···Cg^iv^	1.341 (2)	3.4803 (17)	4.806 (2)	169.96 (13)

Symmetry codes: (i) −*x*+3, −*y*, −*z*+1; (ii) *x*−1, *y*, *z*−1; (iii) −*x*+2, −*y*, −*z*; (iv) *x*, −*y*−1/2, *z*−1/2.

## References

[bb1] Bernstein, J., Davis, R. E., Shimoni, L. & Chang, N.-L. (1995). *Angew. Chem. Int. Ed. Engl.* **34**, 1555–1573.

[bb2] Bruker (2010). *APEX2* and *SAINT.* Bruker AXS Inc., Madison, Wisconsin, USA.

[bb3] Bruno, G. & Randaccio, L. (1980). *Acta Cryst.* B**36**, 1711–1712.

[bb4] Etter, M. C., MacDonald, J. C. & Bernstein, J. (1990). *Acta Cryst.* B**46**, 256–262. 10.1107/s0108768189012929 2344397

[bb5] Farrugia, L. J. (1997). *J. Appl. Cryst.* **30**, 565.

[bb6] Ferguson, G. & Sim, G. A. (1961). *Acta Cryst.* **14**, 1262–1270.

[bb7] Krausse, J. & Dunken, H. (1966). *Acta Cryst.* **20**, 67–73.

[bb8] Macrae, C. F., Edgington, P. R., McCabe, P., Pidcock, E., Shields, G. P., Taylor, R., Towler, M. & van de Streek, J. (2006). *J. Appl. Cryst.* **39**, 453–457.

[bb9] Polito, M., D’Oria, E., Maini, L., Karamertzanis, P. G., Grepioni, F., Braga, D. & Price, S. L. (2008). *CrystEngComm*, **10**, 1848–1854.

[bb10] Sheldrick, G. M. (2008). *Acta Cryst.* A**64**, 112–122. 10.1107/S0108767307043930 18156677

[bb11] Spek, A. L. (2009). *Acta Cryst.* D**65**, 148–155. 10.1107/S090744490804362X PMC2631630 19171970

[bb12] Wilson, C. C., Shankland, N. & Florence, A. J. (1996). *J. Chem. Soc. Faraday Trans.* **92**, 5051–5057.

